# Early warning systems in maternity care: protocol for a qualitative evidence synthesis of maternity care providers’ views and experiences

**DOI:** 10.12688/hrbopenres.13270.1

**Published:** 2021-05-27

**Authors:** Valerie Smith, Kumaresan Cithambaram, Deirdre O'Malley

**Affiliations:** 1 School of Nursing and Midwifery, Trinity College Dublin, Dublin, D02, Ireland; 2 School of Nursing and Midwifery, Queen's University Belfast, Belfast, BT97BL, UK

**Keywords:** Early warning systems, maternity early warning score, MEWS, clinical deterioration, systematic review, qualitative evidence synthesis.

## Abstract

**Background:** Early warning systems (EWS) have been widely adopted for use in maternity settings internationally. The idea in using these systems is early recognition of potential or actual clinical deterioration in pregnant or postpartum women, and escalation of care. Barriers to successful implementation and use of EWS, however, have been identified. If EWS are to be applied consistently, a greater understanding of the views and experiences of EWS from the perspectives of those using and applying EWS in maternity practice is needed. This protocol describes a qualitative evidence synthesis of maternity care providers’ (midwives, obstetricians, and allied maternity care professionals) views and experiences of EWS use and application in practice.

**Methods**: Studies will be included in the review if they report on maternity care providers use and application of EWS in any birth setting. Qualitative studies and studies of mixed methods design, where qualitative data can be extracted separately, will be included. To source relevant literature the electronic databases of MEDLINE, CINHAL, Web of Science Core Collection (incorporating Social Science Citation Index) and Maternity and Infant Care (MIDIRS), from date of inception, will be searched. The methodological quality of the included studies will be appraised using the 12-criteria of the assessment tool developed by the Evidence for Policy and Practice Information and Co-ordinating Centre. Thematic synthesis will be used for synthesising the qualitative data from included studies. The confidence in the findings will be assessed using the Grading of Recommendations Assessment, Development and Evaluation-Confidence in the Evidence from Reviews of Qualitative research.

**Conclusions:** The findings of this qualitative evidence synthesis may provide valuable information on the barriers, challenges, and facilitators for EWS use based on the experiences of those directly involved in EWS application in maternity care provision.

**PROSPERO registration:** CRD42021235137 (08/04/2021)

## Introduction

Early warning systems (EWS) have been introduced in clinical care as a means of formalising the measurement of physiological variables (temperature, pulse rate, blood pressure, respiratory rate, alert status, etc.). Measurements are recorded and collectively assessed, generally using either a points-based scoring system (0, 1, 2, 3, etc.) or a colour-coded traffic light system (red, orange, green) based on each parameter measuring within or outside of agreed physiological norms ^1^. The idea in using these systems is early recognition of potential or actual clinical deterioration in pregnant or postpartum women, and escalation of care. Reports spanning more than a decade from professional bodies and other national maternity clinical guidelines recommend using EWS to identify the potential for clinical deterioration in women who are pregnant or postpartum ^2– 7^. These systems have now been widely adopted for use in maternity settings internationally ^8– 10^.

Although limited, some evidence exists which suggests that use of EWS may be useful in predicting maternal morbidity ^11– 13^, although less so for mortality ^11, 14^. To affect accurate prediction, however, compliance in using EWS consistently in practice and according to recommended schedules, which may vary depending on location and clinical scenario (e.g. 1-hourly during labour, 4-hourly during routine postnatal care, etc.) is required. Studies have demonstrated significant variance in compliance rates with EWS use, ranging from below 50% to 100% across studies ^13, 15– 19^. Compliance in use also appears to diminish as a woman’s length of stay in hospital extends. For example, Allman ^15^ noted that non-recording of observations on EWS ranged from 64% at two hours to 2% at seven hours. Helme ^20^ also reported an increased rate of ‘poor’ recordings on EWS, from 11% at one-hour post-surgery to 27% at two hours and 91% between three and 24 hours postoperatively. Of further concern, 40% of maternity EWS scores in one audit were reportedly inaccurate ^21^.

Barriers to successful implementation and use of EWS have been identified. These include overlap with other charts, staff shortages, lack of training in EWS use, lack of support for EWS, delegating observation recording to maternity care assistants, too time consuming and a lack of validation ^21– 24^. If EWS is to be applied consistently in maternity care for the purpose of early detection and management of clinical deterioration there needs to be enhanced understandings of the views and experiences of EWS from the perspectives of those applying EWS in practice. For this reason, we plan to perform a synthesis of qualitative data of maternity care providers’ views and experiences of maternity EWS.

## Aim

This paper describes a protocol for a qualitative evidence synthesis of maternity care providers’ (midwives, obstetricians, and allied maternity care professionals) views and experiences of EWS use and application in practice. The proposed review is registered with PROSPERO (CRD42021235137, 8 ^th^ April 2021) and adheres to the Preferred Reporting Items for Systematic review and Meta-Analysis Protocols ( PRISMA-P) reporting guidelines ^25^.

## Methods

### Inclusion criteria

We use the SPIDER (sample, phenomenon of interest, design, evaluation, and research type) acronym ^26^ to define the criteria for including studies in the review as follows:

- **S**ample: Maternity care providers (MCPs) involved in caring for pregnant/postpartum women, of low and high risk, and in any birth setting. MCPs are defined as professionally qualified MCP, for example, midwife nurse, doctor, obstetrician and other allied MCPs).
- **P**henomenon of **I**nterest: EWS application/use in clinical practice
- **D**esign: Qualitative studies of any design. Mixed methods studies, where the qualitative data are accessible separately, will also be considered for inclusion. Survey designs with open-ended response options that collect qualitative data may be considered for inclusion. The depth of qualitative data must be sufficient however, for surveys to be included; that is, surveys that report qualitative data in the form of exemplar quotes to support quantitative ‘counts’ or where the data are briefly summarised rather than being formally analysed using a structured approach (e.g. thematic analysis), will be excluded.
- **E**valuation: Inductive themes representative of MCPs views and experiences of EWS use in clinical practice
- **R**esearch type: Published and unpublished studies reporting qualitative data on MCPs views or experiences of EWS application or use.

### Search & selection methods

We will search the electronic databases systematically to identify and retrieve primary research studies. The databases that will be searched are MEDLINE, CINHAL, Web of Science Core Collection (incorporating Social Science Citation Index) and Maternity and Infant Care (MIDIRS). Databases will be searched from the date of inception to the date the search is implemented. Search terms were developed using elements of the SPIDER acronym ( Table 1) and adapted as appropriate for the different databases. Language filters will not be applied to the search, although we will only include studies published in English. The rationale for searching all languages unfiltered is to identify potentially eligible non-English publications, and, depending on how many might be retrieved, whether possible language bias could be introduced by their exclusion. Records retrieved from the databases will be downloaded to Endnote (version EN20). Duplicate records will be removed and the remaining records uploaded to Covidence software ( www.covidence.org) for eligibility screening. Screening records against the review’s eligibility criteria will be undertaken by two members of the review team (VS & KC). All records will be initially screened based on their title and abstract. Those that appear eligible at this level will be forwarded for full text review. Following a review of full-text papers, studies meeting our inclusion criteria will be included in the review. Any uncertainty or disagreements regarding the inclusion or exclusion of records will be resolved through discussion, or, if needed, the record will be assessed by a third review author (DOM) until a consensus decision is achieved. 

**Table 1.** Search terms.

**S**	Nurse* OR midwi* OR (public health nurse*) OR (practice nurse*) OR doctor* OR (general practitioner*) OR obstetrician* OR clinician OR physician OR (healthcare provider) OR (healthcare professional) AND
**PI**	(Physiological track and trigger*) OR (early warning*) OR (early warning NEXT-1 (score OR tool, OR signs OR triggers OR system)) OR mews OR meows OR moews OR (escalation adj protocol*) (escalation adj policy) AND Maternity (MeSH) OR Obstetrics (MeSH) OR Pregnant* OR Pregnancy (MeSH) OR (antenatal OR prenatal OR perinatal OR puerperal OR puerperium OR postnatal OR postpartum OR peripartum OR post-natal OR post-partum OR ante-natal OR ante-partum OR obstetric*).tw OR Postpartum period (MeSH) AND
**E** or **R**	experiences OR experience OR view* OR perceptions OR perception OR voices OR narratives OR qualitative OR (mixed ADJ method) OR (grounded theory) OR phenomenology OR (action research)

We will expand our search by additionally searching the reference lists of studies identified for inclusion in the review and by searching the proceedings of international maternity care conferences (e.g. International Confederation of Midwives Triennial Conference 2017; Normal Labour and Birth Research Conference 2020). We will perform searches of grey literature databases (e.g. https://www.greylit.org/) and will search the reference lists of any identified national clinical guidelines on maternity EWS for potentially relevant studies that might not have been captured in our electronic database search.

### Quality appraisal of included studies

Assessing the methodological quality of included studies formally is a key component of systematic reviews ^27, 28^, and perhaps even more-so in qualitative evidence synthesis as the process of assessment itself can facilitate a deeper understating of the included studies ^29^. A variety of appraisal tools are available for assessing the methodological quality of qualitative studies although these can range from having very broad criteria to consisting of explicit checklists ^30– 32^. For purposes of quality appraisal in this review we have chosen the appraisal tool developed and previously used ^30^ by the Evidence for Policy and Practice Information and Co-ordinating (EPPI) Centre. The tool consists of 12 quality assessment criteria (A-L) across three core domains that are centred on study reporting, data collection and analysis, and study methods ( Table 2). Two pairs of reviewers (VS & DOM; VS & KC) will independently assess the extent to which each quality criterion is met in each study. Irrespective of how many or how few quality criteria are met, we will include all studies for data extraction and synthesis purposes as qualitative studies of poor methodological may still provide important views data that could have considerable relevance to our synthesis. As Thorne advises, eliminating a study based on its fit with particular quality appraisal guidelines “ *may obscure a germ of possibility that, if used to interrogate the reports of other studies, could have led to important new angles of consideration”* ^33^ ^, p.7^.

**Table 2.** Quality appraisal criteria ^30^.

***Quality of the study reporting***	A= Aims and objectives clearly reported B= Adequately described the context of the research C= Adequately described the sample and sampling methods D= Adequately described the data collection methods E= Adequately described the data analysis methods
***There was good or some attempt to establish the***	F= Reliability of the data collection tools G= Validity of the data collection tools H= Reliability of the data analysis I= Validity of the data analysis
***Quality of the methods***	J= Used the appropriate data collection methods to allow for expression of views K= Used the appropriate methods for ensuring the analysis was grounded in the views L= Actively involved the participants in the design and conduct of the study

### Data extraction and synthesis

Data extraction will be based on the aim of the review and will involve extracting the following information:

- Year the study was published
- Aim of the study
- Funding details (if provided)
- Description of the participants and the study setting
- Study duration/timeframes
- Description of the maternity EWS (where provided)
- Method(s) of data collection and analysis
- Findings related to providers’ views and experiences of EWS application and use in maternity care

Using a standardised data extraction form ^25^, the data will be extracted independently by two reviewers followed by accuracy cross-checks. Thematic synthesis, as described by Thomas and Harden for synthesising data from qualitative studies ^34^ will be used to synthesis the studies’ data. In using this method, the following will be undertaken:

i)    Manual line by line coding of extracted data; the extracted text including relevant participant quotes will be reviewed and coded by one member of the review team.

ii)    Descriptive themes will be identified by assessing similarities and differences between codes which will be clustered into descriptive themes. Although one member of the review team will identify the descriptive themes, the review team will meet to discuss these themes to ensure they are reflective of the codes before progressing the synthesis to developing the analytical themes. 

iii)    Generate the analytical (or dominant) themes and sub-themes from the descriptive themes. One member of the review team will generate the analytical themes, however, all members of the review team will be involved in a process of reflection, iteration, and discussion in determining the final themes to ensure they are collectively representative of the studies’ data. This process will enhance synthesis rigour and transparency.

### Assessment of confidence in the review findings; GRADE-CERQual

Grading of Recommendations Assessment, Development and Evaluation-Confidence in the Evidence from Reviews of Qualitative research (GRADE-CERQual) ^35– 40^ will be used to assess levels of confidence in the review findings. Using GRADE-CERQual, distinct review findings are assessed on the components of methodological limitations, coherence, extant or adequacy of contributing data and relevancy to the review question. An overall assessment of High, Moderate, Low or Very Low confidence in each finding, based on the component ratings, is then made ^36^. For purposes of the assessments, we have established *a priori* downgrading criteria as illustrated in Table 3. Judgements are based on an initial assumption of ‘High confidence’ in all findings, and then downgraded accordingly. GRADE-CERQual assessments will be performed by two reviewers independently, as recommended ^35^, with overall confidence levels based on discussions and consensus. 

**Table 3.** GRADE-CERQual downgrading criteria.

GRADE	Downgrading criteria
High	– No or minor concerns in all four components – Moderate concerns in one component and no concerns in remaining three, or minor concerns in one other component
Moderate	– Moderate concerns in one component, minor concerns in two components, and no concerns in remaining component – Moderate concerns in two components and no concerns in remaining two components
Low	– Moderate concerns in two components and minor concerns in at least one other component – Moderate concerns in three components and no concerns in remaining component – Severe concerns in one component and no concerns in remaining three components, or minor concerns in one other component
Very Low	– Moderate concerns in three components and minor concerns in remaining component – Moderate concerns in all four components – Severe concerns in one component and moderate concerns in at least one other component

## Discussion

The findings of this QES will provide valuable insight and understanding of maternity care providers’ views and experiences of EWS application and use in maternity care practice. This evidence may prove valuable for identifying barriers, challenges and facilitators for EWS in practice based on the experiences of those directly involved in using EWS as part of maternity care provision.

In disseminating the findings of the QES, the target audience will be primarily maternity care providers and local or national guideline or policy developers. For this reason, we aim to publish the findings in a healthcare journal that predominantly publishes maternity care research and is known to have a wide maternity care provider readership. Summary findings in the form of short reports will be prepared and shared with national guideline groups (e.g. National Clinical Effectiveness Committee, Ireland), and will be made available to practice development departments in maternity settings. Links to the published report will be shared on social media (Twitter and Facebook), and summary findings distributed via online maternity/midwifery email lists, as appropriate.

## Review status

The search strategy has been implemented. Screening (level 1, title and abstract) the retrieved records for eligibility is currently in progress.

## Data availability

### Underlying data

No data are associated with this article.

### Extended data

Open Science Framework: Early Warning Systems in maternity care: a qualitative evidence synthesis of maternity care providers’ views and experiences. https://doi.org/10.17605/OSF.IO/KBHU5 ^25^.

This project contains the following extended data:

- Template Data Extraction Form.docx

### Reporting Guidelines

Open Science Framework: PRISMA-P Checklist for ‘Early Warning Systems in maternity care: Protocol for a qualitative evidence synthesis of maternity care providers’ views and experiences’: https://doi.org/10.17605/OSF.IO/KBHU5 ^25^.

Data are available under the terms of the Creative Commons Attribution 4.0 International license (CC-BY 4.0).
