# Supplementary material for: Early warning systems in maternity care: protocol for a qualitative evidence synthesis of maternity care providers’ views and experiences
Source: HRB Open Res. 2021 May 27;4:59. [Version 1] doi: 10.12688/hrbopenres.13270.1 (PMC8733824; doi:10.12688/hrbopenres.13270.1)
Supplement: Peer review history

# Peer review: Reviewer response for version 1

**DOI:** 10.21956/hrbopenres.14443.r31044

**Published:** 2022-01-05

**Authors:** Mirjam Lukasse

**Affiliations:** 1 Department of Nursing and Health Promotion, Oslo Metropolitan University, Oslo, Norway; 2 Department of Health and Social Sciences, University of South-Eastern Norway, Vestfold, Norway

This is a study protocol for a qualitative evidence synthesis of maternity care providers' views on and experiences with a type of screening tool to identify a deterioration in a patient's physical condition as early as possible, called an Early Warning System (EWS).

 To date, a synthesis of the qualitative evidence has not been published while there are a number of original research papers in this field. So I am pleased these researchers are willing to take on the work to perform this synthesis. I find the proposal well written. The authors show they know the field well. The proposed methodology is solid. I look forward to reading the synthesis.

 I have just a couple of minor questions. In the methods part of the abstract it is written that studies will be included from the date of inception. Quite a few readers will not know when this was, and this date may also very from country to country. Could this phrase be replaced by a year that ensures all studies are included?

 The authors will only include studies published in English, but report on how many they find in other languages. There may of course not be any papers in other languages. However, I would like the authors to consider having the papers translated if they find one of two in another language. Good information may be lost by not doing this. I realize that English is the dominant language for publication of research. However, midwives who may investigate the introduction of an early warning system in their practice environment may not be such advanced researchers, but can still produce good research under supervision while doing, for example, a Master in Midwifery. If such a study is published in French or German it would be very sad not to have it included. However, this is just a suggestion.

Is the study design appropriate for the research question?

Yes

Is the rationale for, and objectives of, the study clearly described?

Yes

Are sufficient details of the methods provided to allow replication by others?

Yes

Are the datasets clearly presented in a useable and accessible format?

Yes

Reviewer Expertise:

Midwifery and midwifery education

I confirm that I have read this submission and believe that I have an appropriate level of expertise to confirm that it is of an acceptable scientific standard.

# Peer review: Reviewer response for version 1

**DOI:** 10.21956/hrbopenres.14443.r30302

**Published:** 2021-10-19

**Authors:** Ruth Endacott

**Affiliations:** 1 MEDICINE, NURSING AND HEALTH SCIENCES, Monash University, Melbourne, VIC, Australia

The proposed qualitative evidence addresses an important, timely, and under-reviewed area of clinical practice - experience of maternity care providers in the use of Early Warning Systems (EWS). Existing evidence in non-maternity health systems emphasises the impact of human factors on decisions about use of EWS and implementation of escalation. This is a comprehensive, appropriately referenced, protocol for a qualitative evidence synthesis. The rationale is clear and review processes are mostly transparent.  A few minor changes would make the methods more transparent and replicable for future researchers:

 1. Some of the evidence might lie in medical education literature, for example use of EWS in simulated situations. EWS is a key part of education for obstetric emergencies and some studies include qualitative interviews or photo elicitation interviews so might be a valuable source. It is not clear whether these studies are excluded.

 2. Under Methods, it would be helpful to use 'eligibility criteria' as the sub-heading, and then address inclusion criteria and exclusion criteria (i.e. when you have applied your inclusion criteria, what studies would you then exclude?). 

 3. A word is missing in the following sentence: *we will include all studies for data extraction and synthesis purposes as qualitative studies of poor methodological may still provide...*

 4. It would be helpful to add a sentence or two to outline how you will link the thematic synthesis and the output from the GRADECERQual process. This is important for replication of your method but also to ensure that clinicians can easily apply (or not) the QES findings to clinical practice, education of maternity care providers and future policy guidance for EWS in maternity services.

Is the study design appropriate for the research question?

Yes

Is the rationale for, and objectives of, the study clearly described?

Yes

Are sufficient details of the methods provided to allow replication by others?

Partly

Are the datasets clearly presented in a useable and accessible format?

Yes

Reviewer Expertise:

managing deterioration

I confirm that I have read this submission and believe that I have an appropriate level of expertise to confirm that it is of an acceptable scientific standard.
